# Modified gradual ulnar lengthening for treatment of Masada type IIb forearm deformity in children with hereditary multiple osteochondromas

**DOI:** 10.3389/fped.2023.1166255

**Published:** 2023-05-31

**Authors:** Jingyi Fan, Xuejun Zhang, Lin Sun, Chengxin Li, Xinyu Qi, Baosheng Sun

**Affiliations:** Orthopedic Department, Beijing Children's Hospital Affiliated to Capital Medical University, Beijing, China

**Keywords:** modified gradual ulnar lengthening, hereditary multiple osteochondromas, masada type IIb, forearm deformity, ilizarov ring frame, follow-up

## Abstract

**Objective:**

To investigate the effect of modified gradual ulnar lengthening in the treatment of Masada type IIb forearm deformity in children with hereditary multiple osteochondromas (HMO).

**Patients and methods:**

From May 2015 to October 2020, 12 children with Masada type IIb forearm deformity caused by HMO underwent modified gradual ulnar lengthening in our hospital. Clinical and imaging data were retrospectively analyzed. Clinical evaluation included wrist flexion and extension, wrist ulnar and radial deviation, forearm pronation and supination, and elbow range of motion. The radiographic parameters measured included the radial articular angle, carpal slip, and relative ulnar shortening.

**Results:**

The mean operative age of the 12 patients (9 male, 3 female) was 8.5 ± 2.7 years, the mean follow-up was 31.5 ± 5.7 months, and the mean ulnar lengthening was 43.3 ± 9.9 mm. There was no significant difference in the radial articular angle between the preoperative period and the last follow-up (from 36.5° ± 9.2° to 33.8° ± 5.1°, *p* > 0.05). However, significant changes were found in carpal slip (from 61.3% ± 18.8% to 33.8% ± 20.8%) and relative ulnar shortening (from 5.8 ± 3.5 mm to −0.9 ± 4.85 mm) (*p* < 0.05). The range of motion significantly improved after modified gradual ulnar lengthening, including wrist flexion (from 38.3° ± 6.2° to 55.8° ± 9.0°), wrist extension (from 45.0° ± 9.8° to 61.7° ± 8.1°), wrist ulnar deviation (from 41.3° ± 8.6° to 29.6° ± 7.8°), wrist radial deviation (from 18.3° ± 6.2° to 30.0° ± 5.6°), forearm pronation (from 44.6° ± 7.2° to 62.1° ± 8.6°), forearm supination (from 50.0° ± 7.1° to 52.9° ± 6.6°), and elbow range of motion (from 117.1° ± 10.1° to 127.9° ± 5.4°) (all *p* < 0.05). During follow-up, there was one case of needle tract infection and one case of bone nonunion.

**Conclusion:**

Modified gradual ulnar lengthening can effectively treat Masada type IIb forearm deformity caused by HMO and improve forearm function.

## 1. Introduction

Hereditary multiple osteochondromas (HMO) is a hereditary autosomal dominant disease that can occur in childhood, often resulting in severe forearm malformation. Its reported incidence is about 1 in 50,000 (1). The osteochondromas involve the proximal and distal epiphyses of the ulna, especially the distal ulna, causing longitudinal growth disorder of the ulna. This leads to a series of forearm deformities such as ulnar shortening, ulnar curvature, distal radioulnar dislocation, ulnar deviation of the wrist joint, and radial head dislocation, which seriously affect the function of the wrist joint, elbow joint, and forearm (1).

Based on the distribution of the osteochondromas and the severity of forearm deformity, Masada et al. (2) proposed a classical classification that has been widely recognized. However, the treatment of HMO remains controversial. In the present study, we analyzed 12 patients with HMO-induced Masada type IIb forearm deformity who underwent surgical correction by modified gradual ulnar lengthening based on Ilizarov ring external frame fixation and ulnar osteotomy and were followed up.

## 2. Patients and methods

### 2.1. Patients

We retrospectively analyzed 12 young patients with HMO-induced Masada type IIb forearm deformity who were admitted to the Department of Orthopedics of Beijing Children's Hospital from May 2015 to October 2020 (Table 1). All patients were treated by modified gradual ulnar lengthening. The patients comprised nine male and three female children, and six left sides and six right sides were affected. The mean operative age was 8.5 ± 2.7 years, the mean follow-up period was 31.5 ± 5.7 months, and the mean ulnar lengthening was 43.3 ± 9.9 mm.

**Table 1:** Patients’ characteristics.

Patient	Sex	Age (year)	Side	M type	Or	Ul (mm)	EFT (day)	TARF
1	M	8	R	Ⅱb	Y	45	118	Palster
2	F	7	L	Ⅱb	Y	48	133	Brace
3	M	10	R	Ⅱb	N	30	143	Palster
4	F	6	R	Ⅱb	N	36	141	Palster
5	M	10	R	Ⅱb	Y	67	267	Palster
6	F	6	R	Ⅱb	N	40	127	Palster
7	M	14	L	Ⅱb	N	54	143	Brace
8	M	13	R	Ⅱb	Y	39	149	Palster
9	M	6	L	Ⅱb	N	36	135	Palster
10	M	7	L	Ⅱb	N	46	100	Brace
11	F	8	L	Ⅱb	Y	42	144	Palster
12	M	7	L	Ⅱb	N	36	106	Palster

M type, Masada type; Or, osteochondroma resection; Ul, ulnar lengthening; EFT, external fixation time; TARF, treatment after removal of fixation.

### 2.2. Modified gradual ulnar lengthening

We divided the gradual ulnar lengthening procedure into two steps. The first step mainly involved moving the radial head down to promote its reduction, and the second step mainly involved moving the distal ulna down to promote reduction of the distal radioulnar joint and improve wrist function. The patients and their parents were instructed to train the patients' fingers, wrists, forearms, and elbows before surgery to facilitate postoperative functional training. The Illizarov ring frame was adapted in advance according to the length, diameter, and deformity of the patient's forearm.

#### 2.2.1. Step 1

The ulna was extended and the radial head was lowered to facilitate reduction of the dislocated radial head and promote improvement of the humeroradial relationship. After induction of general anesthesia, the Ilizarov ring frame was adjusted and prepositioned on the affected limb so that the distal and proximal rings were located near and far from the radius, respectively, and the elbow and wrist joint movements were checked to ensure that they were not restricted by the frame. Next, 1.5- to 1.8-mm K-wires were used to fix the ulnar and radial joints at the distal part; only the ulna was fixed at the proximal part. After application of tension using a compression and tensioner device, the ulna was locked on the annular frame. The classic four-ring structure could be selected when fitting.

**Figure 1:**
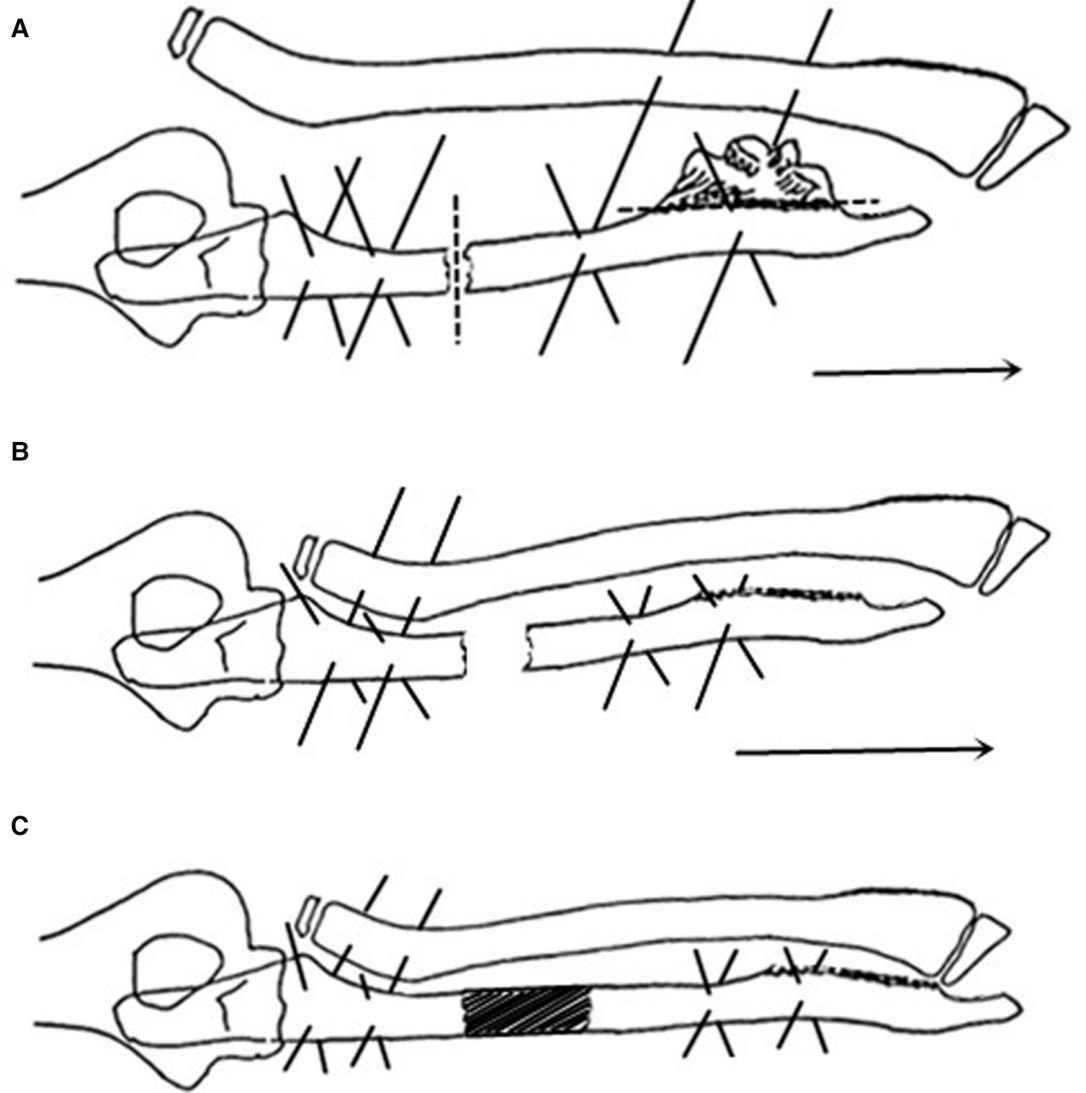
(**A**) The ilizarov frame and K-wire were used to fix the proximal ulna, and combined fixation was performed on the distal radius (the solid line indicates the location of the K-wire). The distal ulnar osteochondroma was selectively cut off, and ulnar osteotomy was performed in the medial-proximal segment (the dotted line indicates the location of the osteotomy). After surgery, the ulna was gradually lengthened to the distal end (direction indicated by arrow), and the dislocated radial head was reduced. (**B**) The K-wire was adjusted to fix the distal ulna, and combined fixation was performed on the proximal ulna and radius (the solid line indicates the K-wire position). After surgery, the ulna was extended further to the distal end (direction indicated by arrow), and the distal radioulnar joint was reduced. (**C**) After distal radioulnar joint reduction, the Ilizarov frame was indwelled to the osteotomy end of the ulna (the solid line indicates the position of the K-wires), and the frame could be removed.

A subperiosteal ulnar osteotomy was performed at the medial and proximal part of the ulna. For patients with a large osteochondroma at the distal part of the ulna that affected the lengthening of the ulna, the osteochondroma was resected (Figure 1A). Ulnar lengthening was performed 7 days after surgery, with a daily lengthening of 1 mm (four times per day, 0.25 mm every 6 h). Anterolateral radiographs of the ulna were taken weekly, and the second operation was performed after the radial head was reduced or lowered to the normal level. In addition, elbow, wrist, and finger functional training was strengthened during the lengthening process to avoid joint movement restriction. At the same time, attention was paid to the occurrence of neurovascular complications during the operation and lengthening to avoid irreversible damage.

#### 2.2.2. Step 2

The distal ulna was moved down to promote distal radioulnar joint reduction and functional recovery of the wrist. After imaging confirmed that the radial head had been reduced or lowered to the normal level, Ilizarov ring frame adjustment surgery was performed. The two K-wires connecting the distal radioulnar joint were removed under anesthesia, one or two K-wires were crossed to fix the distal part of the ulna, and the proximal ulnar fixation pins were adjusted to fix the proximal part of the ulna and radius (Figure 1B). After adjustment, ulnar lengthening was continued as described above until the distal radioulnar joint was reduced. After completion of the two-step lengthening procedure, the Ilizarov ring frame was kept in place until the osteotomy had healed (Figure 1C). After removal of the Ilizarov frame, the limb was placed in a cast. The cast was removed 2 to 3 weeks later for rehabilitation training of the wrist, forearm, and elbow.

### 2.3. Clinical and imaging evaluation

Anteroposterior and lateral radiographs of the affected limb were taken at each follow-up examination before and after surgery to facilitate classification of the forearm deformity and the measurement of imaging parameters. We generally conduct x-ray examination once a week, and reduce it to once every 3–4 weeks after the lengthening. All 12 patients were evaluated by measuring the radial articular angle (RAA), carpal slip (CS), and relative ulnar shortening (RUS) as proposed by Fogel et al. (3) before surgery and at the last follow-up (Table 2). Additionally, wrist flexion and extension, wrist ulnar and radial deviation, forearm pronation and supination, and elbow range of motion of all children were recorded before surgery and at the last follow-up (Table 3) to understand the functional improvement of the affected limb.

**Table 2:** Radiological parameters preoperatively and at last follow-up.

Patient	RAA (°)		CS (%)		RUS (mm)	
	Preoperative	Last follow-up	Preoperative	Last follow-up	Preoperative	Last follow-up
1	33.6	28.4	40	25	3.9	−6.8
2	31.1	32.3	50	10	7.8	−10.6
3	39.6	35.6	65	40	4.1	−3.3
4	36.4	30.2	50	25	4.7	3.5
5	35.8	37	80	35	6	−5.3
6	25.1	24.2	50	50	4.8	−0.8
7	61.5	39.8	90	80	1.2	0.2
8	33.4	36.4	40	15	2.8	0.2
9	41.4	39.7	90	60	6.3	4.6
10	39.4	38.9	80	20	14.6	5.9
11	30.7	28.5	50	15	9.1	0.6
12	29.9	34.3	50	30	4.3	1.2

RAA, radial articular angle; CS, carpal slip; RUS, relative ulnar shortening.

**Table 3:** Range of motion preoperatively and at last follow-up.

Patient	Wrist								Forearm				Elbow	
	Flexion		Extension		Ulnar deviation		Radial deviation		Pronation		Supination		Range of motion	
	PO	LF	PO	LF	PO	LF	PO	LF	PO	LF	PO	LF	PO	LF
1	45	60	55	65	30	20	25	35	55	75	50	55	110	125
2	40	55	50	60	40	30	20	30	50	70	60	60	120	130
3	40	50	45	55	45	35	20	30	45	60	45	50	125	135
4	45	65	50	65	35	25	25	35	45	65	50	50	115	125
5	30	55	35	70	50	30	10	30	40	60	45	50	95	115
6	35	45	55	60	35	30	20	25	45	50	55	60	115	130
7	30	40	30	45	55	45	10	20	35	50	40	45	105	125
8	45	65	55	70	30	20	25	35	55	70	55	55	115	125
9	30	45	30	50	50	40	10	20	35	50	40	40	125	130
10	35	60	35	65	50	30	15	35	35	60	45	50	130	135
11	40	65	50	70	40	20	15	35	45	70	55	60	125	130
12	45	65	50	65	35	30	25	30	50	65	60	60	125	130

The unit of measurement for all data is degrees (°). PO, preoperative; LF, last follow-up.

### 2.4. Statistical analysis

SPSS 20.0 (IBM Corp., Armonk, NY, USA) was used for the statistical analysis. The Kolmogorov–Smirnov test was used to evaluate the normality of all variables. RUS, RAA, wrist flexion, wrist ulnar deviation, forearm pronation and supination, and elbow range of motion were evaluated using a paired t-test. CS, wrist extension, and wrist radial deviation were evaluated by the Wilcoxon test. A *p* value of <0.05 was considered statistically significant.

## 3. Results

**Figure 2:**
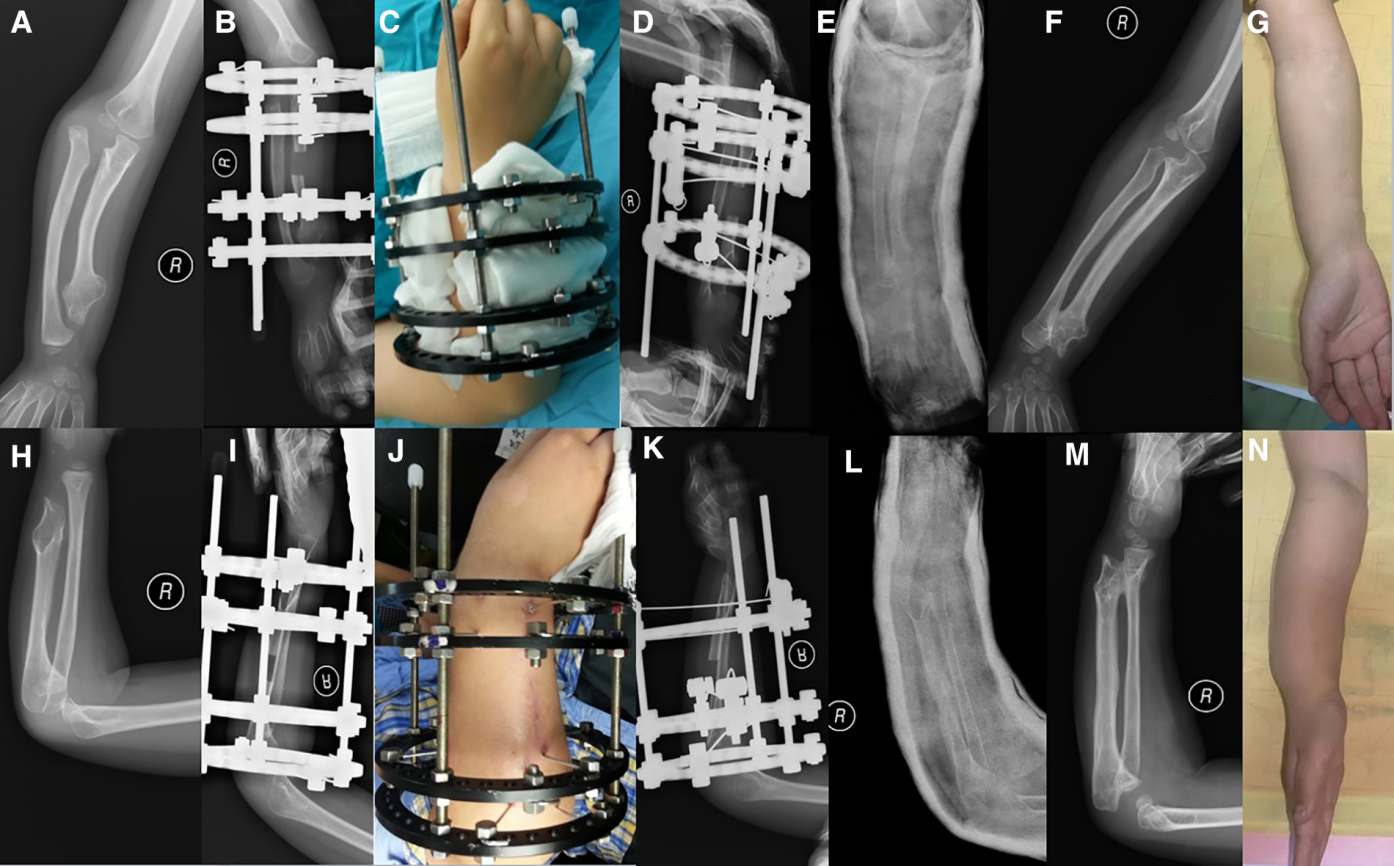
Coronal and sagittal views of forearm of patient 6. (**A,H**) Before surgery. (**C**) After surgery. (**B,I,J**) After Step 1 of lengthening (**D,K**) At the beginning of Step 2. (**E,L**) Plaster fixation after removal of Ilizarov frame. (**F,G,M,N**) Last follow-up.

The 12 young patients (male:female ratio, 3:1; left:right ratio, 1:1) had a mean age of 8.5 ± 2.7 years at the time of the operation, mean ulnar lengthening of 43.3 ± 9.9 mm, a mean Ilizarov ring frame retention time of 142.2 ± 42.3 days, and a mean follow-up time of 31.5 ± 5.7 months. There was no significant difference in the RAA between the preoperative period and the last follow-up (from 36.5° ± 9.2° to 33.8° ± 5.1°, *p* > 0.05). However, significant changes were noted in CS (from 61.3% ± 18.8% to 33.8% ± 20.8%) and RUS (from 5.8 ± 3.5 mm to −0.9 ± 4.85 mm) (*p* < 0.05). The range of motion significantly improved after modified gradual ulnar lengthening, including wrist flexion (from 38.3° ± 6.2° to 55.8° ± 9.0°), wrist extension (from 45.0° ± 9.8° to 61.7° ± 8.1°), wrist ulnar deviation (from 41.3° ± 8.6° to 29.6° ± 7.8°), wrist radial deviation (from 18.3° ± 6.2° to 30.0° ± 5.6°), forearm pronation (from 44.6° ± 7.2° to 62.1° ± 8.6°), forearm supination (from 50.0° ± 7.1° to 52.9° ± 6.6°), and elbow range of motion (from 117.1° ± 10.1° to 127.9° ± 5.4°) (*p* < 0.05 for all) (Table 4). Needle tract infection occurred in one child after surgery and was controlled by oral antibiotic treatment. After removal of the Ilizarov frame, one child was found to have bone nonunion; therefore, the plaster fixation time was increased to 3 months until union was achieved. No intraoperative or postoperative vascular or neurological complications occurred in any patients. Representative cases are shown in Figure 2.

**Table 4:** Clinical and radiological parameters.

Parameters	Preoperative	Last follow-up	*t*/*z* value*	*p* value
**Radiological:**				
RAA	36.5 ± 9.2	33.8 ± 5.1	1.391	0.192
CS (%)	61.3 ± 18.8	33.8 ± 20.8	−2.940*	0.003
RUS	5.8 ± 3.5	−0.9 ± 4.85	4.430	0.001
**Clinical:**				
Wrist flexion (°)	38.3 ± 6.2	55.8 ± 9.0	−10.383	<0.001
Wrist extension (°)	45.0 ± 9.8	61.7 ± 8.1	−3.077*	0.002
Wrist ulnar deviation (°)	41.3 ± 8.6	29.6 ± 7.8	7.532	<0.001
Wrist radial deviation (°)	18.3 ± 6.2	30.0 ± 5.6	−3.134*	0.002
Forearm pronation (°)	44.6 ± 7.2	62.1 ± 8.6	−11.153	<0.001
Forearm supination (°)	50.0 ± 7.1	52.9 ± 6.6	−3.924	0.002
Elbow range of motion(°)	117.1 ± 10.1	127.9 ± 5.4	−6.734	<0.001

Data are presented as mean ± standard deviation.

RAA, radial articular angle; CS, carpal slip; RUS, relative ulnar shortening.

* Nonparametric Wilcoxon test for *z* values.

## 4. Discussion

About 70% of HMO is associated with forearm malformation (4). This is a special clinical manifestation caused by tumor invasion of the proximal and distal ends of the ulna, especially the distal epiphysis of the ulna, resulting in forearm growth disorder. Some studies showed that although the tumor invaded the metaphysis of the ulna and radius, affecting the growth of the forearm, the ulna was more seriously involved than the radius because of the anatomical differences between the ulna and radius. This resulted in a series of deformities such as ulnar shortening, distal radioulnar joint dislocation, radial curvature, radial head dislocation, and ulnar deviation of the wrist joint (5). The treatment of HMO has been controversial among the academic community, and the traditional surgical method (6) has been rejected by most scholars and patients. In recent years, many scholars have reported various methods of lengthening ulnar osteotomy to treat forearm deformities caused by HMO, and certain clinical therapeutic effects have been achieved (7–18). However, different scholars hold different views on the optimal choice of the extension method. The classical extension theory of distraction osteogenesis was first proposed by Ilizarov, and the use of this technique for ulnar extension, downward displacement of the dislocated radial head, and reduction of the distal radioulnar joint has been proven effective (9, 19, 20). In the present study, the Ilizarov ring frame was used for fixation, and the middle part of the ulna was treated by osteotomy and step-by-step lengthening. Meanwhile, the Masada IIb forearm deformity was treated by selective excision according to the size of the distal ulnar tumor and whether the extension was obstructed, and a good effect was obtained. In contrast to simple ulnar lengthening, the step-by-step lengthening method gradually achieves displacement and reduction of the dislocated radial head by adjusting the fixed site of the K-wire and the annular frame, thus recovering the function of the wrist, forearm, and elbow. At the same time, the unique ring structure of the Ilizarov device also ensures good linearity and stability during the lengthening and displacement. Some scholars have reported that pathological changes occurred to the dislocated radial head with the progression of the deformity, and even with the traction and downward movement, the radial head could not be adequately reduced to restore the brachioradial relationship (5, 9, 21, 22). This phenomenon was also observed in the present study. In patients with severe dislocation of the radial head and morphological changes of the ulna and radius, it may be difficult to obtain good brachioradial alignment even if the radial head is pulled down to a normal level; fortunately, however, this does not affect the improvement in the function and appearance of the elbow and forearm. This may be related to the design of the multi-step surgery. The first step in ulnar lengthening is simultaneous fixation of the distal radius. Extending the ulna while lowering the radius as a whole provides more reliable and effective traction than simply lengthening the ulna with indirect traction using soft tissues such as the distal radioulnar joint and interosseous membrane. Contracture of the soft tissue and fascia tissue achieves better extension. Moderate ulnar extension can facilitate good recovery of the distal radioulnar joint and correction of ulnar deviation deformity of the wrist joint (5), and the extension in the second step also provides strong tension for the ulna and promotes good reduction of the distal radioulnar joint. However, we also have concerns similar to those of the above scholars; namely, a series of pathological changes and the growth and development of the radial head after dislocation may still bring great uncertainty to the stability of the brachioradial–radial relationship and the stability of the radial head. Thus, long-term observation and further study are needed. In the Masada classification, types I and II are characterized by obvious exoplastic tumor lesions at the distal end of the ulna. Accurate evaluation should be performed before or during the ulnar osteotomy extension procedure. For patients with large tumors that have affected forearm function or may hinder extension, tumor resection should be performed in advance or at the same time. Some reports have described the treatment of forearm deformity through simple tumor resection (3, 10). All patients in our study had Masada type IIb forearm deformities, and five of them underwent resection of osteochondromas distal to the ulna. The expected results were obtained from the perspective of the lengthening procedure and postoperative forearm function.

Measurement of RUS, RAA, and CS was proposed by Fogel et al. (3) to evaluate the imaging data of the patient. Through a comparative study between the preoperative and final follow-up data, we found that the changes in RUS were most direct and that the distal shortening of the ulna due to dysplasia was corrected through the gradual extension the ulna, especially in the second step. Mild overextension is recommended to prevent recurrence of the deformity, and studies have indicated that excessive extension of 5 mm may be more appropriate (20). In the present study, the lengthening of the ulna and the restoration of the distal ulnar and radioulnar relationship also improved the ulnar deformity of the wrist due to the length mismatch between the ulna and radius, resulting in significant improvement in CS before surgery and at the last follow-up. However, we did not see significant recovery of the RAA because of the small short-term effect of ulnar lengthening alone on the radius morphology. This may be related to the fact that the curvature of the radius cannot be significantly improved by the above treatment, requiring longer follow-up and observation. It had been reported that the wrist deformities caused by HMO could be corrected by using growth modulation technique which could significantly improve RAA in a short period of time (23). But this technique was only used for the correction of distal forearm deformities and could not achieve the reduction of the radial head. In addition, according to the follow-up results, gradual ulnar lengthening can significantly improve the wrist and forearm functions, so the growth modulation technique was not applied in this study.

The Ilizarov ring frame provides linear, stable, and continuous retractor force in the process of ulnar extension, but the following six points require attention during the application process. First, an appropriate aperture ring should be selected according to the length, diameter, and degree of deformity of the forearm. A too-large or too-small ring may cause obstacles to joint activity or extension. Second, during K-wire fixation and ulnar osteotomy dissection, attention should be paid to avoid repeated operations, which may cause excessive soft tissue injury and bleeding, leading to interfascial compartment syndrome. At the same time, attention should be paid to avoid long-term high-speed drilling, which may cause physical damage to adjacent blood vessels and nerves. Third, attention should be paid to ensure that the diameter of the ring is suitable for the patient's arm and that the angle between each crossing K-wire is greater than 60° to ensure stability. The limbs should be placed in the center of the ring when drilling in to avoid the difficulty of needle care due to reduction of the frame size, which may lead to skin and soft tissue complications. Fourth, on the premise of ensuring fixed strength, K-wires with a small diameter should be used as far as possible to reduce soft tissue irritation, infection, and other complications. Fifth, attention should be paid to control the speed of the extension process. Too-fast or too-slow extension may lead to bone nonunion or premature healing; therefore, radiographic examinations should be regularly performed during the extension process to determine whether this has occurred. Sixth, flexion and extension training of the wrist joint, metacarpophalangeal joint, and interphalangeal joint should be strengthened in the process of ulnar extension to avoid joint flexion and extension limitations after extension. We noted that Cho's study used a similar external fixation strategy, but with a larger diameter internal fixation system and bilateral cortical bone fixation which may cause implantation difficulties and severe soft tissue stimulation, especially at the distal ulna (20). Therefore, we prefer to use K-wire with smaller diameter which can reduce soft tissue stimulation and cross fixation which can provide more stable internal fixation. Even though, we still worried about that so many K-wires that are not well tolerated in children and will also look at this question in the future.

Different researchers have different views on the selection of age for surgery. Arms et al. (5) stated that although surgery could improve the appearance and imaging, it could not significantly improve the function of the forearm; thus, they recommended that the age at surgery should not be too young. Some scholars have also proposed that for patients with low bone maturity, the deformity may recur after orthopedic surgery; therefore, downward displacement of the radial head and ulnar lengthening should be performed when the bone is more than 10 years old or nearly mature (18, 24). We believe that if the diagnosis is clear and forearm deformity is obvious, early ulnar lengthening should be performed to promote radial head reduction; improve wrist, forearm, and elbow function; and avoid ulnar shortening, radial head dislocation, and further exacerbation of both morphological changes.

With respect to device selection, the Orthofix orbital single-arm extension exoframe has been used to treat HMO-induced forearm deformity in many studies with a good curative effect (7–9, 12, 16). The Ilizarov ring frame was used in all patients in the present study. Compared with the single-arm track frame, the technical difficulty of placement is relatively low, the clinical operation is easy, the collateral damage is small, and it is easier to maintain linearity and stability during the extension process. Because of the small diameter of the K-wire, the incidence of complications such as nail tract infection is relatively low, and there are no strict requirements regarding the structure and strength of the placement site. These advantages are particularly important in cases of severe distal ulnar dysplasia or decreased local bone structure strength after luminal resection.

In terms of control of the lengthening rate, we followed the classic Ilizarov theory of traction osteogenesis and tissue formation in the present study. The lengthening began immediately after the formation of osteotylus (callus covering the ends of the fractured bone) in the early stage, generally 1 week after surgery, and was then extended by 1 mm every day. Too-fast or too-slow lengthening would have led to complications. Some foreign scholars have reported that one-stage extension can be used for one-time extension of ulnar shortening within 2 cm (3, 25), but we believe that this technique has certain risks and that gradual extension can reduce the occurrence of complications.

Regular examination is very necessary during the lengthening. Although the dose was controlled within a safe range, we should try to evaluate bone regeneration by using examinations such as ultrasound in future evaluation to reduce radiation exposure in children.

In the present study, one patient developed mild needle tract infection 6 weeks after stopping prolongation because of improper care by his family members. The infection manifested as increased needle tract secretions, scabbing, mild swelling of the surrounding soft tissues, and mild pain, and the condition was controlled by 1 week of therapy with oral cefuroxime axetil tablets (0.25 g twice daily). Radiographs of the forearm of another patient showed that the osteotomy end was healed, but the amount of callus was small. Re-examination after removal of the Ilizarov ring frame showed that the actual healing of the ulna was poor, and bone nonunion was suspected; therefore, the cast was fixed for 3 months, a large posterior callus formed, and the plaster was removed after self-healing. Although there have been literature reports on bone nonunion after ulnar extension and bone grafting (26), this patient's nonunion healed after conservative treatment for a period of time, and he did not undergo bone grafting. No serious neurological or vascular complications occurred during the operation or during the prolongation.

The main innovation point of this study was to use Ilizarov frame and K-wire to fix the distal and proximal ends of ulna step by step to improve the method of gradual ulna lengthening, which ensures the effect of the radial head reduction while avoiding excessive distal movement of the radial head. Meanwhile, this new method can resist the interosseous membrane stretching to promote the distal reduction of the ulna effectively.

The small number of enrolled cases and the lack of effective control studies are the main limitations of this manuscript, which need to be further improved in our subsequent studies.

## 5. Conclusion

In summary, the modified ulnar progressive lengthening method based on ulnar osteotomy and Ilizarov external frame fixation is an effective method for the treatment of Masada IIb forearm deformity caused by HMO. Through the step-by-step lengthening method, the ulna is gradually lengthened and lowered, promoting reduction of the radial head and radioulnar joint. The function of the elbow joint, wrist joint, and forearm as well as several imaging indexes were improved. Before treatment, it is necessary to clarify the classification of the deformity and establish an accurate surgical plan. During the operation, close attention is needed to avoid the occurrence of side injury, control the speed of the prolongation process, and prevent complications. Considering the progression of the primary disease and the growth and development of the patient, long-term follow-up of relevant imaging parameters and joint function is necessary.

## Data Availability

The original contributions presented in the study are included in the article, further inquiries can be directed to the corresponding author/s.
